# Robotic‐assisted versus laparoscopic nephroureterectomy; a systematic review and meta‐analysis

**DOI:** 10.1002/bco2.208

**Published:** 2023-01-22

**Authors:** Niall J. O'Sullivan, Ailish Naughton, Hugo C. Temperley, Rowan G. Casey

**Affiliations:** ^1^ Department of Urology Tallaght University Hospital Dublin 24 Ireland; ^2^ School of Medicine Trinity College Dublin Dublin 2 Ireland; ^3^ Department of Urology St. Vincent's University Hospital Dublin 4 Ireland; ^4^ Department of Surgery St. James's Hospital Dublin 8 Ireland

**Keywords:** laparoscopy, nephroureterectomy, robotic surgery, upper tract urothelial carcinoma

## Abstract

**Background:**

Upper tract urothelial carcinoma (UTUC) is the malignant transformation of urothelial cells, from the renal calyces to the ureteral orifices. While the benefits of minimally invasive nephroureterectomy over their open counterpart have been established, the optimal technique remains a debate. We aimed to assess current evidence in the literature and compare outcomes between robotic‐assisted (RANU) and laparoscopic nephroureterectomy (LNU).

**Methods:**

A systematic review of the literature was performed for studies comparing RANU and LNU for bladder cancer. Outcome measurements were recurrence rates (local and distal), positive margins, positive lymph node yield and perioperative outcomes. Meta‐analysis was performed using *Review Manager 5*.

**Results:**

Our results demonstrate a significantly higher mortality rate in patients undergoing laparoscopic nephroureterectomy when compared with the robotic‐assisted approach for the treatment of UTUC (1.8% vs. 1.1%, *p* = 0.008); however, these results were inconsistent on sensitivity analysis and should therefore be interpreted with caution. No significant difference was observed for other outcomes.

**Conclusion:**

The ideal approach to minimally invasive radical nephroureterectomy remains undetermined. Future research, ideally prospective randomised studies, should focus on long‐term outcomes, in particular recurrence, recurrence‐free survival, overall survival and the correlation between surgical technique and survival.

## 1 INTRODUCTION

Upper tract urothelial carcinoma (UTUC) is defined as the malignant transformation of urothelial cells lining the urinary tract, from the renal calyces to the ureteral orifices.^1^ UTUC accounts for only 5–10% of transitional cell carcinomas, with the vast majority arising from within the bladder.^2^, ^3^ Unlike bladder tumours, which are generally confined to the bladder mucosa, 60% of UTUCs are muscle invasive at diagnosis.^3^ The gold standard treatment modality for patients presenting with UTUC and a normal contralateral kidney is an open radical nephroureterectomy (RNU), alongside bladder cuff excision (BCE).^4^, ^5^, ^6^

In the management of UTUC, robot‐assisted nephroureterectomy (RANU) is still not widely implemented and data are scarce in the literature. The available data are primarily retrospective and includes only small, single‐institution sample sizes with limited follow‐up.^7^, ^8^, ^9^ The implementation of robot‐assisted laparoscopic nephroureterectomy is increasing, but evidence to support its use is limited, inconsistent and of generally low quality.^10^ The aim of this study is to assess evidence from current randomised controlled trials (RCTs) and observational studies in the literature and compare outcomes between standard laparoscopic nephroureterectomy (LNU) and robot‐assisted laparoscopic nephroureterectomy (RANU).

## 2 METHODS

### 2.1 Study design and reporting guidelines

This study is a systematic review of retrospective cohort studies and follows PRISMA reporting guidelines.

### 2.2 Search strategy

The following databases were searched as part of the systematic review in March 2022: Medline, EMBASE and Web of Science. The following search terms were used: “robotic‐assisted*,” “laparoscopic*,” “nephroureterectomy*,” “urothelial carcinoma.” The symbol “*” was used to allow variations on a word stem to be included in the search results. Furthermore, the following MeSH (medical subject headings) were used: (robotic‐assisted[MeSH] OR laparoscopic[MeSH]) AND (nephroureterectomy[MeSH] OR urothelial carcinoma[MeSH]). The last date of search was 3 March 2022. The grey literature was also searched to further identify ongoing works of literature.

### 2.3 Inclusion criteria

Studies in English assessing the outcomes of robotic‐assisted versus laparoscopic nephroureterectomies were assessed for eligibility based on the following inclusion criteria:

- Study design:
  - Randomised controlled trials
  - Cohort studies
  - Case–control studies
- Participants
  - Patients with urothelial carcinomas undergoing nephroureterectomy
- Intervention:
  - Comparison of robotic‐assisted versus laparoscopic approach
- Outcomes:
  - Primary: Recurrence (overall), positive surgical margins, lymph node yield, severe morbidity (Clavien–Dindo >2)
  - Secondary: Quantitative measure of overall morbidity, operative time, intraoperative complications, transfusions, postoperative length of stay, pathological outcomes, metastasis, mortality, recurrence‐free survival, cancer‐specific survival, correlation between surgical technique and survival
  - Basic participant characteristics: age, sex, primary tumour location, T‐stage and grade

### 2.4 Exclusion criteria

- Study design:
  - Case reports
  - Case series
  - Conference abstracts
- Participants
  - Patients without urothelial malignancy
- Intervention:
  - No comparison of RANU and LNU
  - Hand‐assisted laparoscopic nephroureterectomy
- Outcome:
  - Qualitative measures only
  - Unclear outcomes

### 2.5 Outcomes of interest

- Primary outcomes:
  - The primary aim of this study was to quantitatively assess recurrence, positive surgical margins, lymph node yield and severe morbidity (Clavien–Dindo >2)
- Secondary outcome:
  - The secondary aims of this study were to quantitatively assess overall morbidity, operative time, intraoperative complications, transfusions, postoperative length of stay, pathological outcomes, metastasis, mortality, recurrence‐free survival, cancer‐specific survival and correlation between surgical technique and survival.
- Basic participant characteristics, including age, sex, primary tumour location, T‐stage and grade

### 2.6 Study selection, data extraction and critical appraisal

A database was created using the reference managing software EndNote X9™. Two researchers reviewed outputs from the searches independently of each other.

Initially, duplicates were removed. Study titles were then screened and assessed for potential relevance. The abstracts of selected potential studies were then read and assessed for eligibility for inclusion, based on the inclusion/exclusion criteria detailed above. Rejected studies were grouped together in the database by their reason for exclusion. The full texts of the abstracts deemed eligible for inclusion were then further analysed using the same criteria. Conflicts between the two reviewers were resolved following an open discussion and final decision by the senior author.

In order to extract and store data efficiently, the Cochrane Collaboration screening and data extraction tool, Covidence, was used. Data were collected by two reviewers independently, using the following headings: Study details, study design, population, intervention, comparison groups and outcomes. Conflicts between the two reviewers were resolved following an open discussion and final decision by the senior author.

A critical appraisal of the methodological quality and risk of bias of the included studies was performed. The critical appraisal was completed by two reviewers independently. Quality assessment of nonrandomised controlled trials (non‐RCTs) was performed according to Newcastle–Ottawa Scale (NOS). Furthermore, the certainty of evidence was assessed using the Grading of Recommendations, Assessment, Development and Evaluations (GRADE) tool for grading quality of evidence.^11^

### 2.7 Data analysis

Statistical analysis was performed using Revman Statistical Software (Ver. 5 Copenhagen, Denmark). Binary outcome data were reported as odd ratios (OR) and 95% confidence interval (95% CI) were estimated using the Mantel–Haenszel method. For continuous data, mean differences and 95% CI were estimated using inverse variance weighting. Outcome measures (mean + standard deviation and median + interquartile range) were recorded. If needed, outcome variables (mean and SD) were estimated from the median and range using formula described by Hozo et al.^12^ Heterogeneity was assessed by *I*‐squared statistics, with >50% being considered as considerable heterogeneity. A random effects model was applied in cases of considerable heterogeneity (>50%) and a fixed effects model applied otherwise. Statistical significance was attributed to *p* value <0.05. Sensitivity analysis was performed using Revman Statistical Software (Ver. 5 Copenhagen, Denmark). Consistency of results was determined by excluding studies based on size, chronology and methodological quality.

### 2.8 Systematic review registration

Our systematic review was registered on PROSPERO in February 2022 (ID: 374275).

## 3 RESULTS

### 3.1 Search results

**FIGURE 1:**
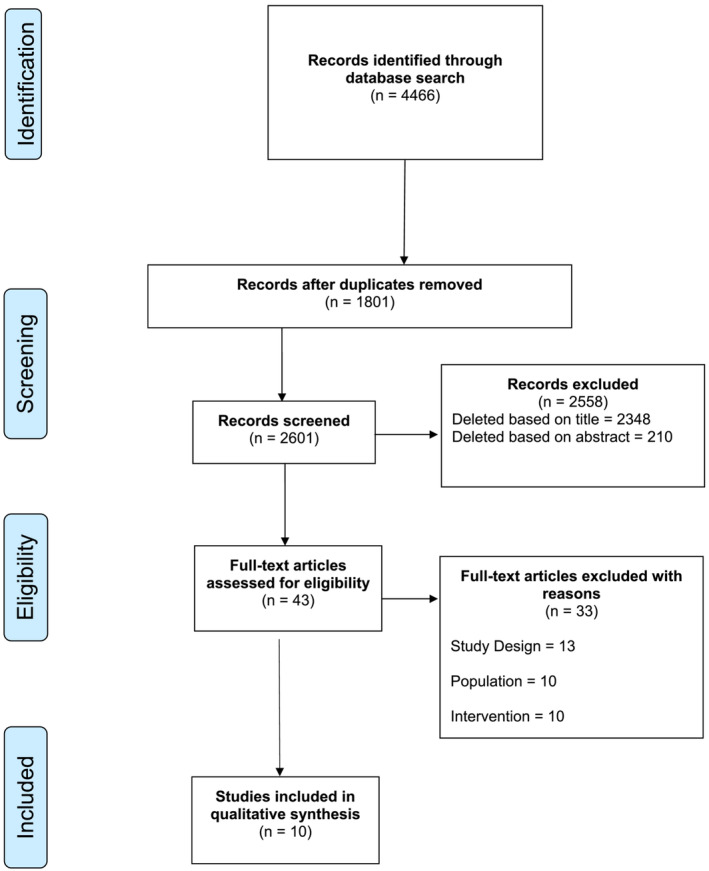
Study selection. A PRISMA Flowchart of the selection of relevant publications included in this review

The literature search described above yielded a total of 4466 results (Figure 1). Following the removal of duplicates, 2601 studies were screened. After the initial screen, 253 abstracts were reviewed and assessed for eligibility, of which 43 were selected for full text review.

From these 43 full texts, 33 were excluded for the following reasons: 13 were an incorrect study design; 10 did not analyse patients with urothelial malignancy; 10 did not compare outcomes of RANU and LNU. A total of 10 studies met the inclusion criteria, all of which were included in the quantitative analysis.

### 3.2 Methodological characteristics and quality of studies

**FIGURE 2:**
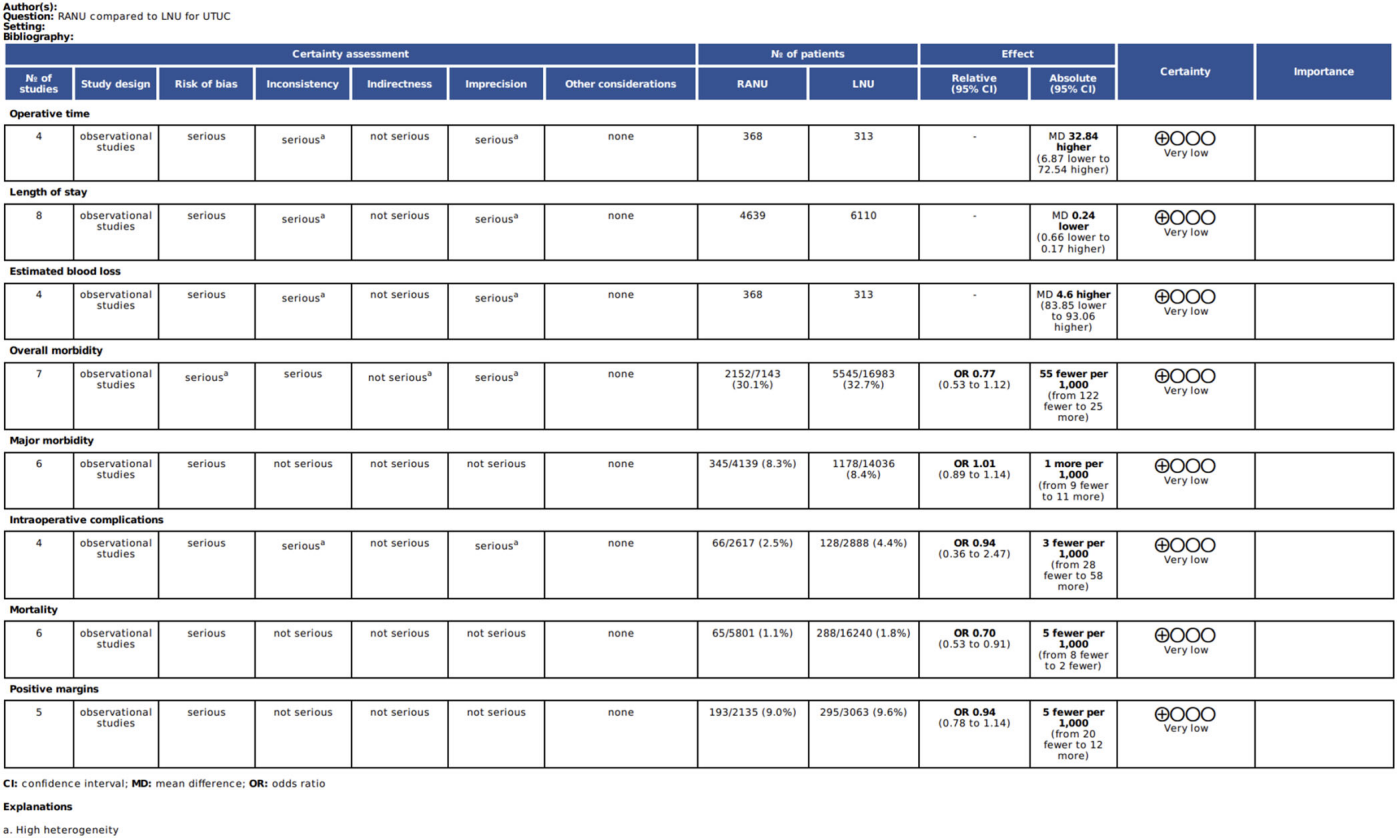
GRADE Certainty of Evidence table

All 10 of the included studies were retrospective cohort studies.^13^, ^14^, ^15^, ^16^, ^17^, ^18^, ^19^, ^20^, ^21^, ^22^ Table 1 summarises the methodological characteristics of the included studies. The methodological quality of the included studies was generally good and is presented in Table 2. Four studies achieved a rating of 7 or higher on the Newcastle Ottawa Scale (NOS), meeting criteria for “high quality” studies. The GRADE certainty of evidence ranged from very low to low and is presented in Figure 2.

**TABLE 1:** Methodological characteristics of the included studies

Study	Year	Country	Study design	Publication journal
Ambani et al.^13^	2014	USA	Retrospective cohort study	*Urology*
Kenigsberg et al.^14^	2021	USA	Retrospective cohort study	*Journal of Endourology*
Lee et al.^15^	2019	USA	Retrospective cohort study	*PLOS One*
Lenis et al.^16^	2018	USA	Retrospective cohort study	*Urologic Oncology*
Li et al.^17^	2021	China	Retrospective cohort study	*Frontiers in Oncology*
Melquist et al.^18^	2016	USA	Retrospective cohort study	*Urology*
Pearce et al.^19^	2016	USA	Retrospective cohort study	*Urologic Oncology*
Tinay et al.^20^	2015	USA	Retrospective cohort study	*BJU International*
Trudeau et al.^21^	2014	Canada	Retrospective cohort study	*Canadian Urological Association Journal*
Veccia et al.^22^	2022	USA	Retrospective cohort study	*Journal of Endourology*

**TABLE 2:** Newcastle Ottawa Scale (NOS) risk of bias assessment for nonrandomised studies

Title	Year	Selection				Comparability	Outcome			Quality
		Representativeness of the exposed cohort	Sample size (<25 = no star)	Nonrespondents	Ascertainment of the exposure	The subjects in different outcome groups are comparable	Assessment of outcome	Statistical test	Period (<4 weeks)	
Ambani	2014	✸	✸	/	✸	✸	/	✸	✸	6
Kenigsberg	2020	✸	✸	✸	✸	✸✸	✸	✸	✸	9
Lee	2018	✸	✸	/	/	✸✸		/	✸	5
Lenis	2017	✸	✸	/	/	✸✸	/	/	/	4
Li	2021	✸	✸	✸	/	/	✸	✸	✸	6
Melquist	2016	✸	✸	✸	/	✸✸	/	✸	✸	7
Pearce	2016	✸	✸	✸	/	✸✸	✸	✸	/	7
Rodriguez	2017	✸	✸	✸	✸	✸	/	✸	✸	7
Tinay	2015	✸	✸	/	/	✸✸	✸	✸	✸	7
Trudeau	2014	✸	✸	✸	✸	✸	✸	✸	✸	8
Veccia	2022	✸	✸	✸	/	/	/	✸	/	4

### 3.3 Participant characteristics

The total number of participants from the 10 included studies was 29 987. Overall, 9175 patients underwent robot‐assisted radical nephroureterectomy, with the remaining 20 812 patients undergoing laparoscopic radical nephroureterectomy. The baseline characteristics of participants are outlined in Table 3. Method of bladder cuff management and number of patients presenting with pT ≥ 3 are outlined in Table 4.

**TABLE 3:** Baseline characteristics of participants

Study	Number of participants			Age (mean ± SD) or [median]		M:F	
	RANU	LNU	Total	RANU	LNU	RANU	LNU
Ambani 2014^13^	22	22	44	70.1 ± 2.2	70.8 ± 2.2	14:8	16:6
Kenigsberg 2021^14^	1129	1502	2631	‐	‐	741:388	909:593
Lee 2019^15^	124	137	261	67.6 ± 11.3	68.6 ± 10.4	85:39	97:40
Lenis 2018^16^	762	1385	2147	70 ± 10.9	70.6 ± 10.4	469:293	772:613
Li 2021^17^	141	458	599	‐	‐	80:61	264:194
Melquist 2016^18^	37	63	100	‐	‐	26:11	36:27
Pearce 2016^19^	2286	2638	4924	‐	‐	‐	‐
Tinay 2015^20^	3774	13 317	17 091	‐	‐	2178:1596	7950:5367
Trudeau 2014^21^	715	1199	1914	73 ± 3	75 ± 2.5	451:264	695:504
Veccia 2022^22^	185	91	276	72 ± 2.1667	71 ± 2.1667	106:179	55:36

**TABLE 4:** Bladder cuff management and *n* pT ≥ 3

Study	Bladder cuff management		pT ≥ 3 *n* (% of total)	
	RANU	LNU	RANU	LNU
Ambani et al.^13^	Pure robotic	Transurethral or open approach	8 (36.4%)	8 (36.4%)
Kenigsberg et al.^14^	‐	‐	223 (19.8%)	330 (22%)
Lee et al.^15^	Pure robotic or open if tumour involving distal ureter or VUJ	Open approach	35 (28.2%)	48 (35%)
Lenis et al.^16^	‐	‐	270 (35.4%)	540 (39%)
Li et al.^17^	‐	‐	54 (38.3%)	171 (37.3%)
Melquist et al.^18^	Pure robotic	Open approach	7 (18.9%)	20 (31.7%)
Pearce et al.^19^	‐	‐	‐	‐
Tinay et al.^20^	‐	‐	‐	‐
Trudeau et al.^21^	‐	‐	‐	‐
Veccia et al.^22^	Multiple techniques	Multiple techniques	21 (11.4%)	17 (18.7%)

### 3.4 Tumour location

Tumour location was reported in six of our included studies.^13^, ^15^, ^16^, ^17^, ^18^, ^22^ Out of 3387 cases, 65.3% (*n* = 2212) of tumours were located within the kidney, 31.2% (*n* = 1056) were within the ureter and the remaining 3.5% (*n* = 119) were involved both the kidney and ureter.

### 3.5 Positive surgical margins

**FIGURE 3:**
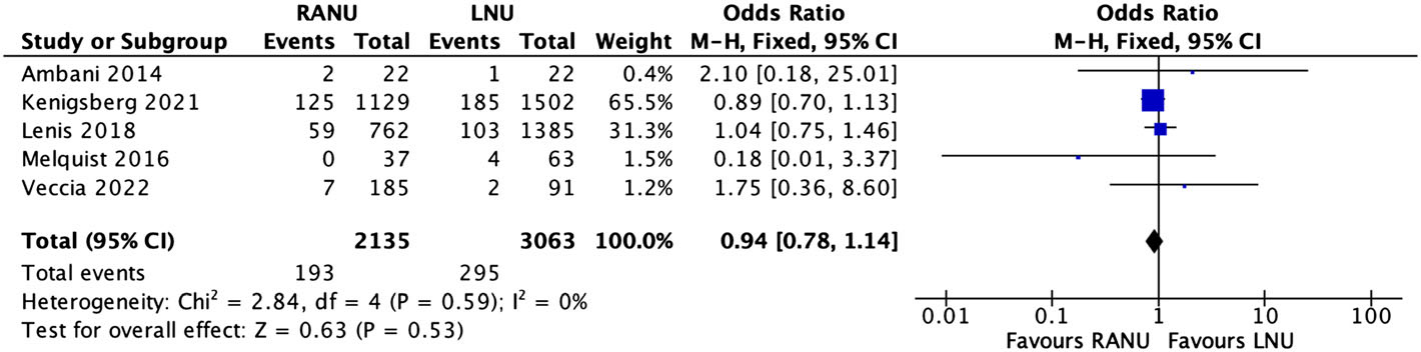
Positive surgical margins meta‐analysis results

Five studies reported the rate of positive surgical margins postnephroureterectomy. The rate was 9% (193/2135) in the RANU group and 9.6% (295/3063) in the LNU group. A meta‐analysis of the included studies using the M‐H fixed‐effects model showed no significant difference between the groups for this outcome (OR 0.94, 95% CI, 0.78–1.14, *p* = 0.53), with no heterogeneity reported across the included studies (*I* ^2^ = 0%) (Figure 3) (very low certainty evidence).

### 3.6 Major morbidity

**FIGURE 4:**
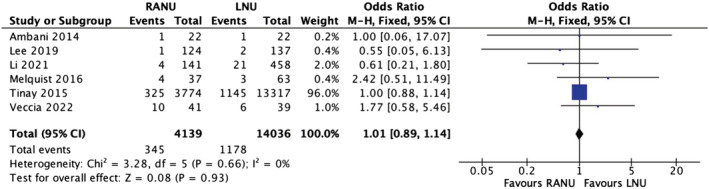
Major morbidity meta‐analysis results

Six studies reported major morbidity rates (Clavien–Dindo >2) between the two groups. The rate was 8.3% (345/4139) in the RANU group and 8.4% (1178/14036) in the LNU group. A meta‐analysis of the included studies using the M‐H fixed‐effects model showed no significant difference between the two groups for this outcome (OR 1.01, 95% CI, 0.89–1.14, *p* = 0.93), with no heterogeneity between the studies (*I* ^2^ = 0%) (Figure 4) (very low certainty evidence).

### 3.7 Overall morbidity

**FIGURE 5:**
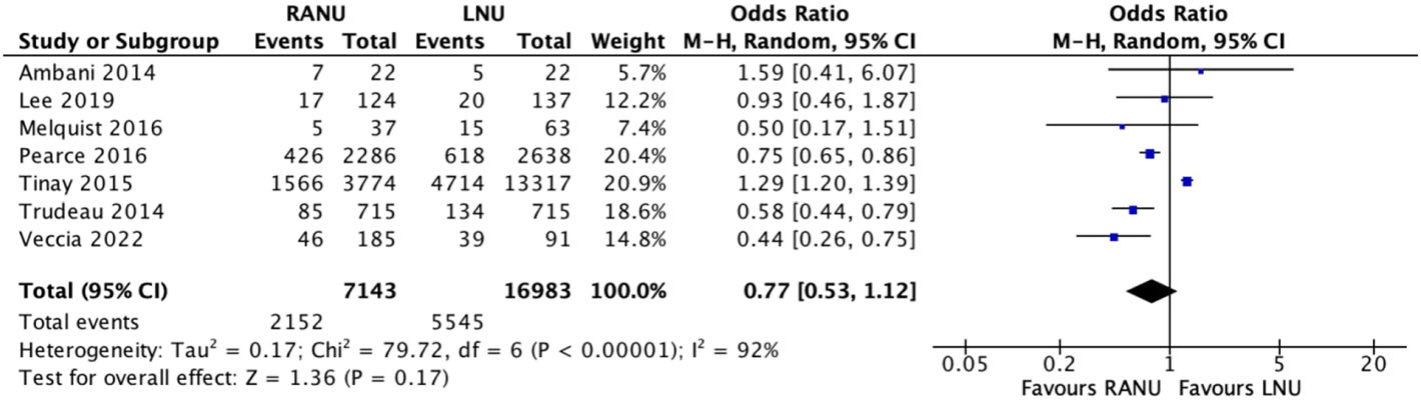
Overall morbidity meta‐analysis results

Seven studies reported overall morbidity rates between the two groups. The morbidity rate was 30.1% (2152/7143) in the RANU group and 32.7% (5545/16983) in the LNU group. A meta‐analysis of the included studies using the M‐H random‐effects model revealed no significant difference between the two groups in terms of overall morbidity (OR 0.77, 95% CI, 0.53–1.12, *p* = 0.17), with considerable heterogeneity between studies (*I* ^2^ = 0%) (Figure 5) (very low certainty evidence).

### 3.8 Operative time

**FIGURE 6:**
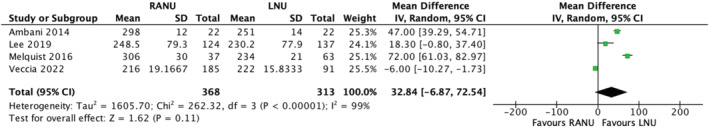
Operative time meta‐analysis results

Four studies reported on operative time (minutes). A meta‐analysis performed using the random‐effects model revealed a reduced operative time in the LNU group; however, these results were not statistically significant (MD 32.84 minutes shorter in the LNU group, 95% CI, −6.87 – 72.54, *p* = 0.11). Considerable heterogeneity was found (*I* ^2^ = 99%) (Figure 6) (very low certainty evidence).

### 3.9 Intraoperative complications

**FIGURE 7:**
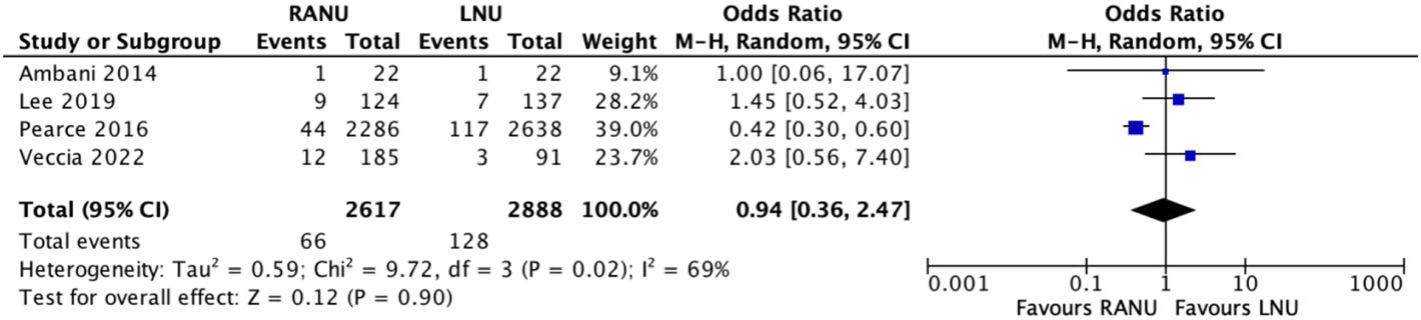
Intraoperative complications meta‐analysis results

Four studies reported on intraoperative complication rates between the two groups. The rate was 2.5% in the RANU group and 4.4% in the LNU group. A meta‐analysis using the M‐H random‐effects model revealed showed no significant difference between the two groups in regards to this outcome (OR 0.94, 95% CI, 0.36–2.47, *p* = 0.90), with considerable heterogeneity between included studies (*I* ^2^ = 69%) (Figure 7) (very low certainty evidence).

### 3.10 Postoperative length of stay

**FIGURE 8:**
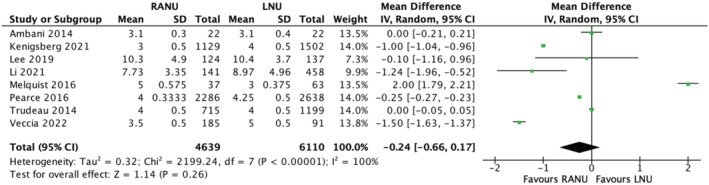
Postoperative length of stay meta‐analysis results

Eight studies reported on postoperative length of stay. A meta‐analysis performed using a random‐effects model revealed a slight reduction in postoperative length of stay in the RANU cohort; however, these results were statistically insignificant (MD 0.24 days shorted in RANU group, 95% CI, −0.66 – 0.17, *p* = 0.26). There was considerable heterogeneity between studies (*I* ^2^ = 100%) (Figure 8) (very low certainty evidence).

### 3.11 Perioperative mortality

**FIGURE 9:**
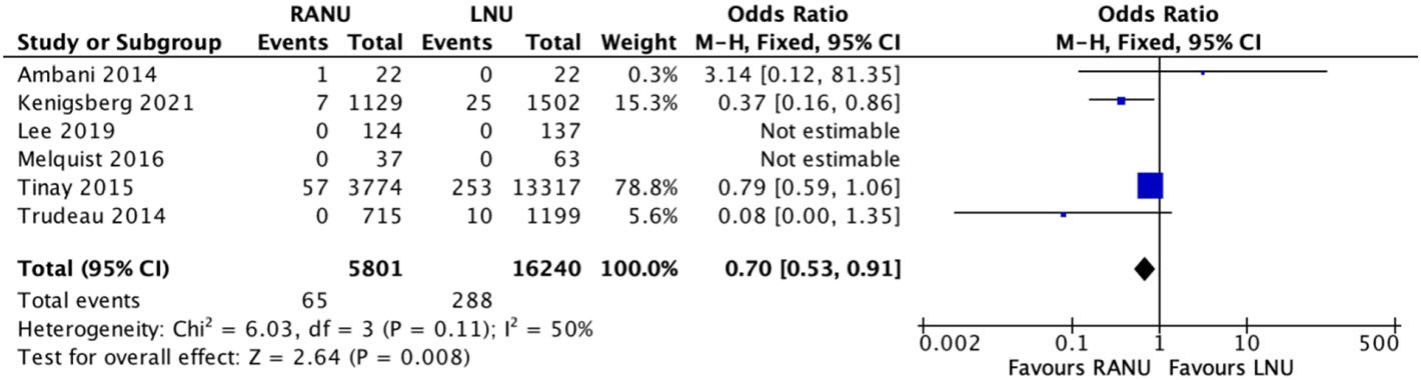
Mortality meta‐analysis results

Six studies reported on 30‐day perioperative mortality rates. The rate was 1.1% (65/5801) in the RANU group and 1.8% (288/16240) in the LNU group. A meta‐analysis of the included studies using the M‐H fixed‐effects model revealed a significant difference between the two groups in regards to mortality rate (OR 0.7, 95% CI, 0.53–0.91, *p* = 0.008), with marginal heterogeneity between studies (*I* ^2^ = 50%) (Figure 9) (very low certainty evidence). It is important to interpret this analysis with caution due to heterogeneity between the measurement of perioperative mortality between studies. One study reported 30‐day mortality rate,^14^ one study reported 90‐day mortality rate,^20^ one study reported “in‐hospital” mortality rate^21^ and the remaining three studies did not specify.^13^, ^15^, ^18^

### 3.12 Estimated blood loss

**FIGURE 10:**
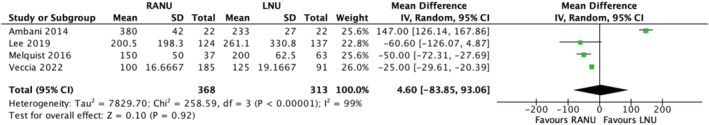
Estimated blood loss meta‐analysis

Estimated blood loss was reported by four studies. A meta‐analysis performed using a random‐effects model revealed no difference between the two groups in regards to blood loss (MD 4.6 ml less in LNU group, 95% CI, −83.85–93.06, *p* = 0.92). There was considerable heterogeneity between studies (*I* ^2^ = 99%) (Figure 10) (very low certainty evidence).

### 3.13 Sensitivity Analysis

A sensitivity analysis for the rate of perioperative mortality was performed to evaluate the stability of the results. Mortality rates proved inconsistent when studies were excluded based on size (OR 0.5, 95% CI, 0.22–1.16, *p* = 0.11), chronology (OR 0.6, 95% CI, 0.29–1.23, *p* = 0.17) and methodological quality (OR 0.5, 95% CI 0.22–1.16, *p* = 0.11). These inconsistencies warrant caution when interpreting the meta‐analysis results for perioperative mortality.

### 3.14 Qualitative outcomes

Unfortunately, due to a lack of data available, we were unable to quantify and meta‐analyse several of our primary and secondary endpoints, including recurrence, lymph node yield, need for transfusion, several pathological outcomes and long‐term survival outcomes. Two of our included studies reported recurrence rates.^13^, ^18^ Ambani et al. reported a recurrence rate (bladder and distant) of 68.1% (*n* = 15) in their RANU group, compared with 54.5% (*n* = 12) in the LNU cohort.^13^ Conversely, Melquist et al. demonstrated a higher recurrence rate in their LNU cohort, when compared with that of RANU, 39.7% (*n =* 9) and 24.3% (*n =* 25), respectively.^18^

## 4 DISCUSSION

Our results demonstrate no clear advantage of one minimally invasive technique over another in treatment of UTUC via nephroureterectomy. With the exception of perioperative mortality, the robotic‐assisted approach provides no clear benefit over the pure laparoscopic approach; however, mortality benefit should be interpreted with caution due to moderate heterogeneity between studies (*I* ^2^ = 50%) and inconsistent results on sensitivity analysis. Ambani et al. were the only study to provide an individual breakdown of cause of death, reporting hematemesis as the aetiology of the single death in their robotic‐assisted cohort.^13^ To our knowledge, this is the most up‐to‐date meta‐analysis comparing the robotic‐assisted and laparoscopic approaches to nephroureterectomy, incorporating several new large studies not previously included in prior meta‐analyses.

Long‐term, prospective studies comparing these outcomes between the robotic‐assisted and laparoscopic approaches have yet to be performed and will undoubtedly provide invaluable information into the optimal surgical approach in the treatment of patients with UTUC. While the benefits of MIS are well established, it is imperative that further research is performed to identify the ideal method to provide patients with the best perioperative and long‐term outcomes possible. Future research, ideally prospective randomised studies, should focus on long‐term outcomes, in particular recurrence, recurrence‐free survival, overall survival and the correlation between surgical technique and survival. In order to achieve the above, the authors have made the following recommendations for future studies comparing the laparoscopic and robotic‐assisted approaches to nephroureterectomy:

- A large prospective controlled study, ideally randomised, comparing laparoscopic nephroureterectomy and robotic‐assisted nephroureterectomy in the treatment of adult patients with upper tract urothelial carcinoma.
- Clear description of method of bladder cuff excision, with a subgroup analysis of oncological outcomes for each method.
- Long‐term data, incorporating a comparison of recurrence, overall survival, disease‐free survival, overall mortality and disease‐specific mortality.
- Quality of life data, via a standardised patient‐reported outcome measures (PROMs) questionnaire.

Our study had several limitations. While the evidence regarding RANU in the treatment of UTUC has increased substantially in recent years, it still remains sparse and of generally low quality. Similarly, a lack of prospective, randomised trials, as well as long‐term data on recurrence, prevented us from analysing this outcome and drawing conclusions on the superiority of one minimally invasive technique over another. Despite these limitations, our review demonstrates no clear benefit of RANU or LNU in terms of perioperative outcomes and provides a good foundation for future research to focus on long‐term outcomes comparing these two techniques.

## 5 CONCLUSION

The ideal approach to minimally invasive radical nephroureterectomy remains undetermined. Future research, ideally prospective randomised studies, should focus on long‐term outcomes, in particular recurrence, recurrence‐free survival, overall survival and the correlation between surgical technique and survival.

## CONFLICTS OF INTEREST

The authors report no conflicts of interest.

## AUTHOR CONTRIBUTIONS

*Data extraction*: Niall J. O'Sullivan, Ailish Naughton, and Hugo C. Temperley. *Data synthesis*: Niall J. O'Sullivan and Ailish Naughton. *Manuscript preparation*: Niall J. O'Sullivan, Ailish Naughton, Hugo C. Temperley, and Rowan G. Casey. *Manuscript writing*: NOS, AN, RC. *Manuscript review/editing*: Niall J. O'Sullivan and Rowan G. Casey.

## Supporting information


**Data S1.** Supporting Information
