# Supplementary material for: Robotic‐assisted versus laparoscopic nephroureterectomy; a systematic review and meta‐analysis
Source: BJUI Compass. 2023 Jan 22;4(3):246–55. doi: 10.1002/bco2.208 (PMC10071076; doi:10.1002/bco2.208)
Supplement: Supplementary file 1 — Data S1. Supporting Information [file BCO2-4-246-s001.docx]

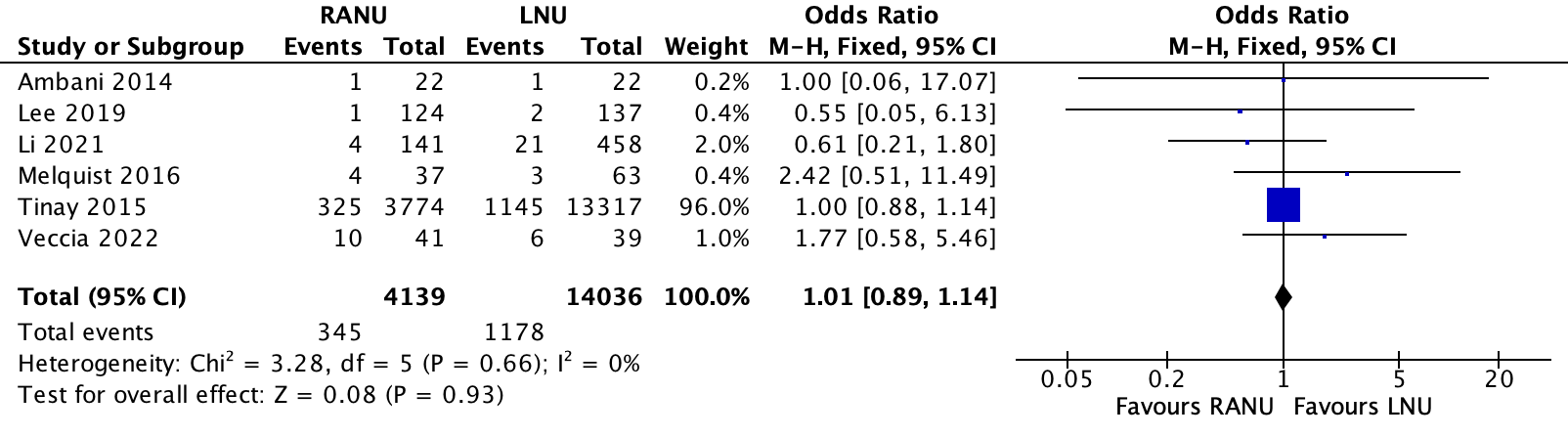


**Figure 4: Major morbidity meta-analysis results**


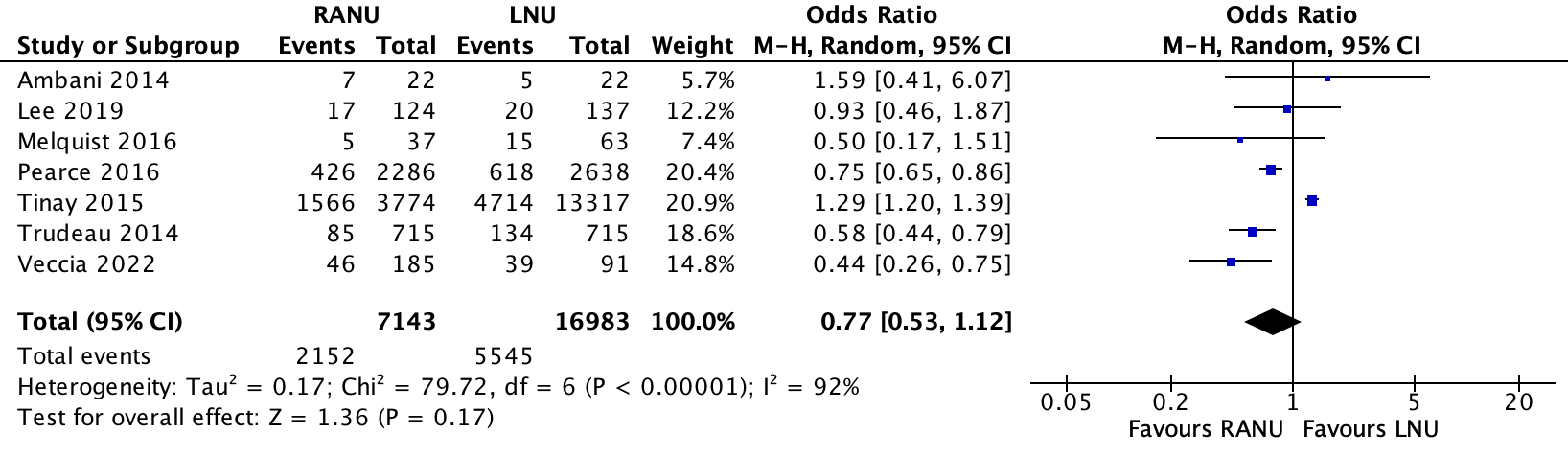


**Figure 5: Overall morbidity meta-analysis results**


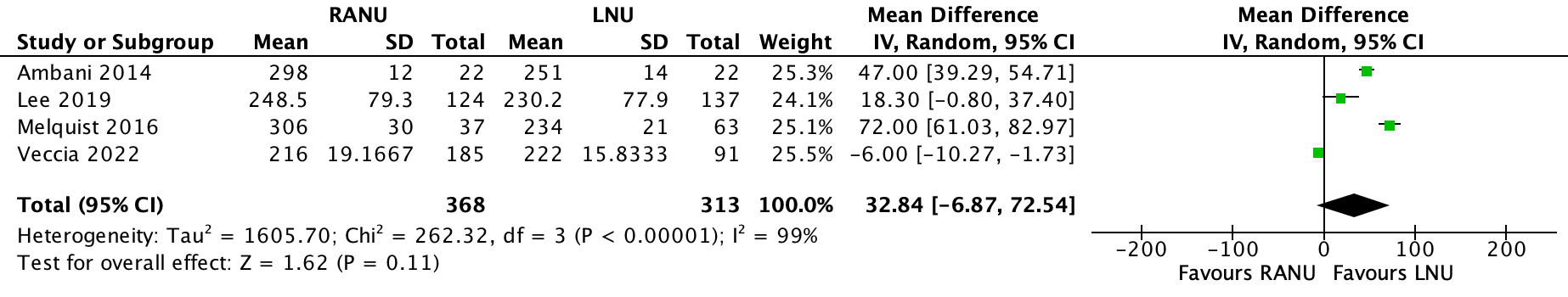


**Figure 6: Operative time meta-analysis results**


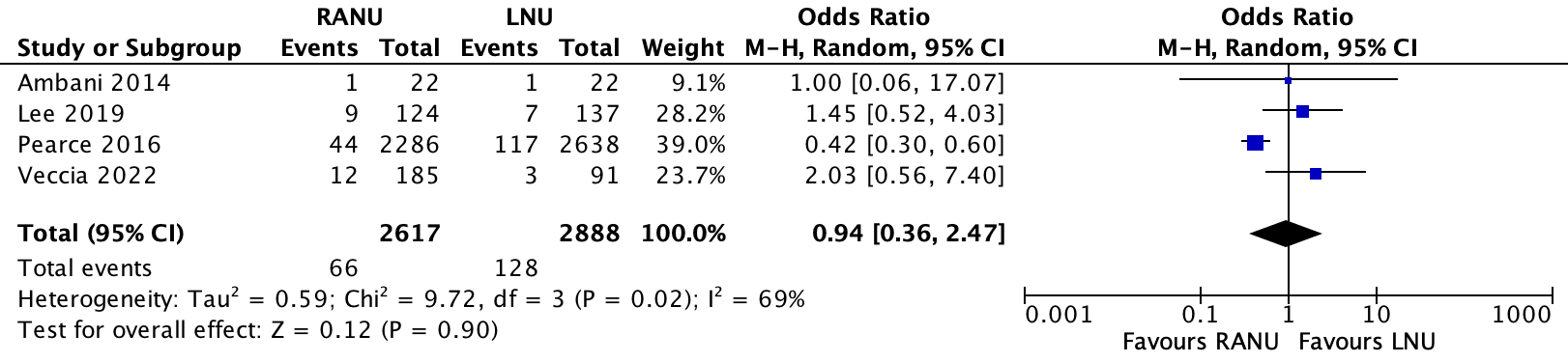


**Figure 7: Intra-operative complications meta-analysis results**

**
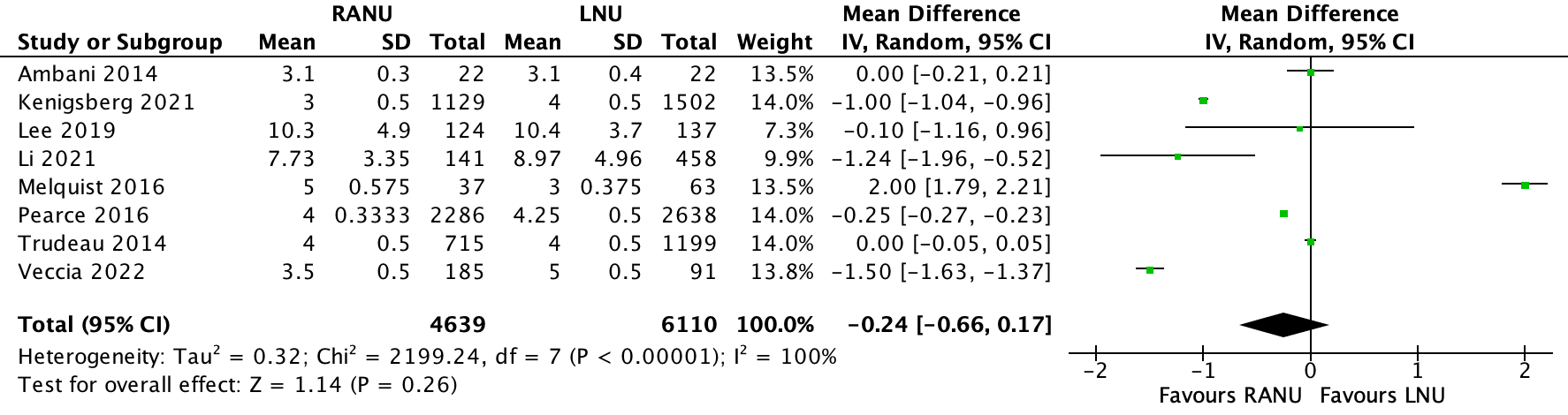
**

**Figure 8: Post-operative length of stay meta-analysis results**

**
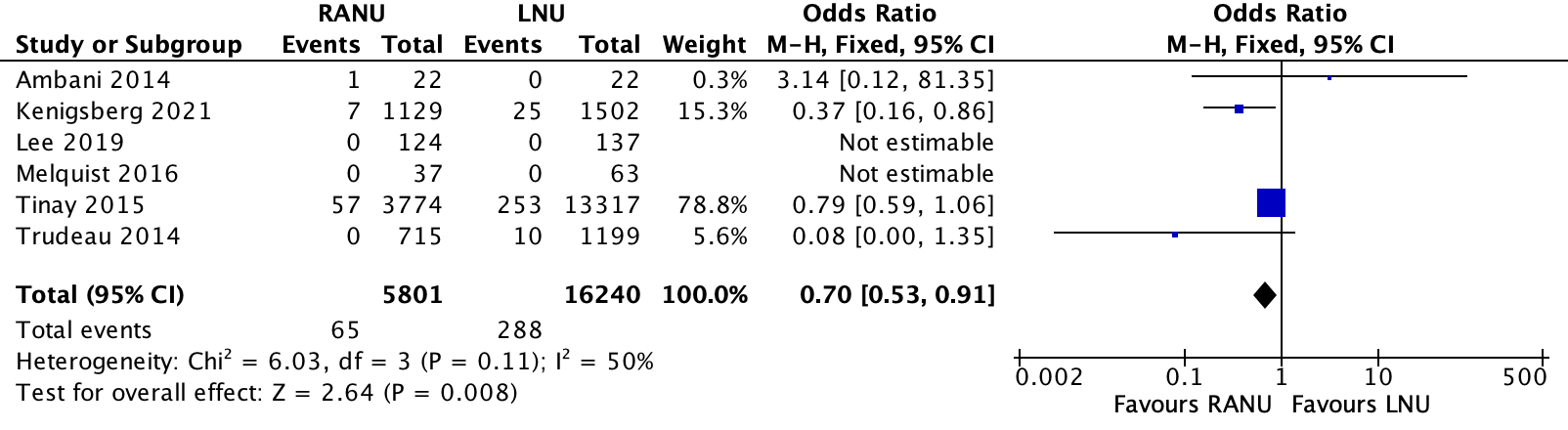
**

**Figure 9: Mortality meta-analysis results**

**
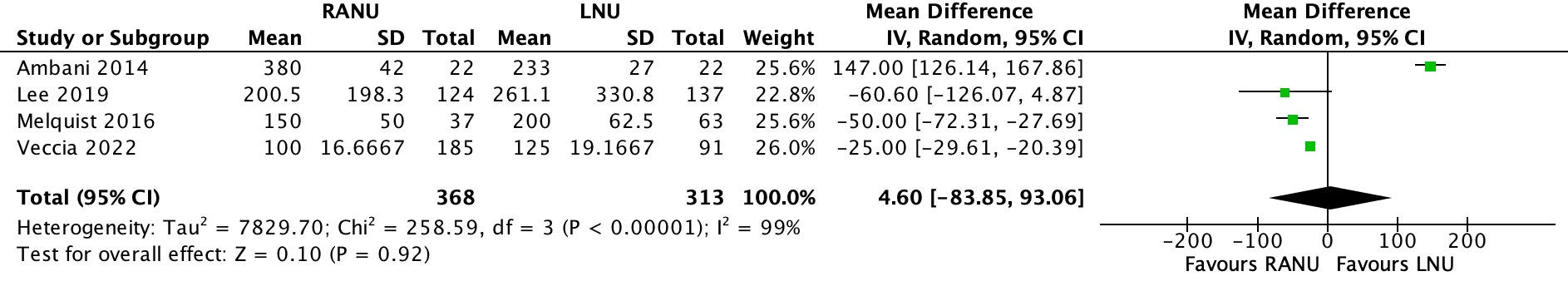
**

**Figure 10: Estimated blood loss meta-analysis**

| **Study** | **Bladder cuff management** | | **pT≥3 *n* (% of total)** | |
| --- | --- | --- | --- | --- |
|  | **RANU** | **LNU** | **RANU** | **LNU** |
| Ambani et al.(28) | Pure robotic | Transurethral or open approach | 8 (36.4%) | 8 (36.4%) |
| Kenigsberg et al.(29) | - | - | 223 (19.8%) | 330 (22%) |
| Lee et al.(30) | Pure robotic or open if tumour involving distal ureter or VUJ | Open approach | 35 (28.2%) | 48 (35%) |
| Lenis et al.(31) | - | - | 270 (35.4%) | 540 (39%) |
| Li et al.(32) | - | - | 54 (38.3%) | 171 (37.3%) |
| Melquist et al.(33) | Pure robotic | Open approach | 7 (18.9%) | 20 (31.7%) |
| Pearce et al.(34) | - | - | - | - |
| Tinay et al.(35) | - | - | - | - |
| Trudeau et al.(36) | - | - | - | - |
| Veccia et al.(14) | Multiple techniques | Multiple techniques | 21 (11.4%) | 17 (18.7%) |

**Table 4: Bladder cuff management and *n* pT≥3**
